# Endotoxin Tolerance Variation over 24 h during Porcine Endotoxemia: Association with Changes in Circulation and Organ Dysfunction

**DOI:** 10.1371/journal.pone.0053221

**Published:** 2013-01-10

**Authors:** Markus Castegren, Paul Skorup, Miklós Lipcsey, Anders Larsson, Jan Sjölin

**Affiliations:** 1 Centre for Clinical Research Sörmland, Uppsala University, Uppsala, Sweden; 2 Section of Infectious Diseases, Department of Medical Sciences, Uppsala University, Uppsala, Sweden; 3 Section of Anesthesiology and Intensive Care, Department of Surgical Sciences, Uppsala University, Uppsala, Sweden; 4 Section of Clinical Chemistry, Department of Medical Sciences, Uppsala University, Uppsala, Sweden; University of Cincinnati, United States of America

## Abstract

Endotoxin tolerance (ET), defined as reduced inflammatory responsiveness to endotoxin challenge following a first encounter with endotoxin, is an extensively studied phenomenon. Although reduced mortality and morbidity in the presence of ET has been demonstrated in animal studies, little is known about the temporal development of ET. Further, in acute respiratory distress syndrome ET correlates to the severity of the disease, suggesting a complicated relation between ET and organ dysfunction. Eighteen pigs were subjected to intensive care and a continuous endotoxin infusion for 24 h with the aim to study the time course of early ET and to relate ET to outcome in organ dysfunction. Three animals served as non-endotoxemic controls. Blood samples for cytokine analyses were taken and physiological variables registered every third hour. Production of TNF-α, IL-6, and IL-10 before and after endotoxin stimulation *ex vivo* was measured. The difference between cytokine values after and before *ex vivo* LPS stimulation (Δ-values) was calculated for all time points. ΔTNF-α was employed as the principal marker of ET and lower ΔTNF-α values were interpreted as higher levels of ET. During endotoxin infusion, there was suppression of *ex vivo* productions of TNF-α and IL-6 but not of IL-10 in comparison with that at 0 h. The *ex vivo* TNF-α values followed another time concentration curve than those *in vivo*. ΔTNF-α was at the lowest already at 6 h, followed by an increase during the ensuing hours. ΔTNF-α at 6 h correlated positively to blood pressure and systemic vascular resistance and negatively to cardiac index at 24 h. In this study a temporal variation of ET was demonstrated that did not follow changes in plasma TNF-α concentrations. Maximal ET occurred early in the course and the higher the ET, the more hyperdynamic the circulation 18 h later.

## Introduction

Endotoxin, a lipopolysaccharide structure of the Gram-negative bacterial cell wall, induces a systemic inflammatory response in animals and humans that has similarities to that seen in sepsis and septic shock [1]. It is known that re-exposure of animals or cells to endotoxin after a previous dose is not accompanied by the profound inflammatory and metabolic changes that are induced by the first encounter [2]. This phenomenon is called endotoxin tolerance and has been widely investigated and reviewed [2]–[4].

Endotoxin tolerance is often demonstrated by a diminished release of tumor necrosis factor-α (TNF-α), but reprogramming of leukocytes and changes in the production of a number of inflammatory mediators are other important features of the changes in the inflammatory response [5]–[7]. In animal studies the presence of endotoxin tolerance has been shown to reduce mortality [5], [6] as well as protect different organs from ischemia/reperfusion injury [7]–[9] and tissues in models of thermal injury and hemorrhagic shock [10], [11]. Underlining the clinical relevance, tolerance to endotoxin has been reported in septic patients, post-operative patients, trauma and pancreatitis patients and patients surviving cardiac arrest and resuscitation [12]. Contrary to the general view of organ protection, the severity of the lung injury in patients with acute respiratory distress syndrome (ARDS) has been demonstrated to be associated with a high level of endotoxin tolerance [13]. Similarly, pediatric patients after cardiopulmonary bypass that showed a maintained endotoxin stimulated production of TNF-α *ex vivo* were less likely to have post-operative complications [14]. Furthermore, the induction of tolerance is complicated as shown by the increased responses observed in some animal models after the second hit of endotoxin [15], [16].

In experimental models the time to induce tolerance, i.e. between the first endotoxin dose and the second hit, varies widely between different studies; in the above referred investigations the first hit was given from 9 h to 6 days before the second hit. Gresiman and Hornick described an early and a late phase of endotoxin tolerance in which the early phase develops and starts to wane within hours and the second phase, associated with high serum levels of anti-endotoxin antibodies, takes several days to evolve [17]. There is not much conclusive work done on the temporal development of the early phase of endotoxin tolerance, as recently discussed by West et al [18]. This naturally raises questions on the reproducibility of and the comparability between diverging results regarding the inflammatory response and different physiologic parameters in different studies. Furthermore, it underscores the need for more knowledge about the time course of endotoxin tolerance and its relation to organ dysfunction.

In the current experiment the principal aim was to study the time course of the early phase of endotoxin tolerance during 24 h in an intensive care model of porcine endotoxemia. The ability of *ex vivo* endotoxin-stimulated blood to produce inflammatory cytokines, mainly TNF-α, was used to assess the degree of endotoxin tolerance. A secondary aim was to investigate whether the level of endotoxin tolerance was related to outcome in terms of circulatory and organ dysfunction parameters.

## Materials and Methods

### Ethics statement

The study, approved by the Animal Ethics Board (Permit number: C 215/5) in Uppsala, Sweden, included 21 healthy pigs with a weight of 28.4 kg (range 25.6–32.8 kg). Water and food access was *ad libitum* until 1 h before the experiment. The pigs were handled in strict accordance to the Guide for the Care and Use of Laboratory Animals (National Institutes of Health). All surgery was performed under balanced general anesthesia and all efforts were made to minimize suffering.

### Anesthesia and preparations

The doses of the anesthetic drugs as well as the anesthetic procedure, preparations and the intensive care protocol have previously been described in detail [19]–[21]. Briefly, all animals were given 50 mg xylazin intramuscularly immediately before transport to the research facility. General anesthesia was induced by injecting a mixture of tiletamin-zolazepam 6 mg×kg^−1^ and xylazin 2.2 mg×kg^−1^ intramuscularly. A bolus dose of morphine 20 mg and ketamine 100 mg was given intravenously (i.v.) immediately before the airway was secured by tracheal intubation. The animals were then mechanically ventilated throughout the experiment. The anesthesia was maintained as a continuous infusion of sodium pentobarbital 8 mg×kg^−1^×h^−1^, morphine 0.48 mg×kg^−1^×h^−1^, ketamine 1 mg×kg^−1^×h^−1^ and pancuronium bromide 0.26 mg×kg^−1^×h^−1^ dissolved in 2.5% glucose solution. Saline 2 mL×kg^−1^×h^−1^ (0.9% sodium chloride solution) was administered i.v., resulting in a total fluid administration rate of 10 mL×kg^−1^×h^−1^. Before the start of surgical preparations, cefuroxime 20 mg×kg^−1^ was administered i.v., after which an arterial line, a central venous line, a 7F Swan-Ganz catheter and a cystostomia catheter were inserted under aseptic conditions. To reduce the risk of intra-abdominal hypertension a 14 Ch Bülow drainage tube was placed in the abdominal cavity before closure of the abdomen. After the preparations were completed, the animals were given a colloid fluid bolus of 4% succinylated gelatin solution, 10 mL×kg^−1^, followed by a 30-min stabilization period before the start of the protocol.

### Intensive care

Initial respiratory settings were: respiratory rate 25 min^−1^, inspiratory-expiratory ratio 1∶2, inspired oxygen fraction (FiO_2_) 0.3, positive end-expiratory pressure (PEEP) 5 cm H_2_O and tidal volume (V_T_) 10 mL×kg^−1^. V_T_ was adjusted before the start of the protocol to result in an arterial partial pressure of carbon dioxide (PaCO_2_) of 5.0–5.5 kPa (38–41 mmHg). The ventilator used was either a Servo 900C™ or Servo I™ (Siemens-Elema, Stockholm, Sweden). Atelectasis was prevented and treated by performing an alveolar recruitment maneuver and changing the animal's body position every third hour.

To resemble an intensive care setting the animals were treated in accordance with a protocol to maintain vital parameters within preset limits. This protocol has been described in detail elsewhere [20]. Briefly, blood gases were reviewed hourly and V_T_ increased or decreased 10% depending on the result to maintain PaCO_2_ within the initial range. FiO_2_ was increased 0.1 or decreased 0.05 to maintain arterial partial pressure of oxygen (PaO_2_) in the range of 12–18 kPa (90–135 mmHg). All other respiratory settings were kept constant during the experiment. If mean arterial pressure (MAP) approximated mean pulmonary arterial pressure (MPAP) during the first 90 min of the protocol, a single dose of adrenaline 0.1 mg was given i.v. This was done to counteract the initial, often quickly reversible pulmonary hypertension found in pigs during initial endotoxemia [22], [23]. If MAP decreased below 50 mmHg at any given time during the experiment, a colloid fluid bolus of 10 mL×kg^−1^ was given with the possibility of an additional colloid fluid bolus of 5 mL×kg^−1^ within the next 60 min if MAP remained below 50 mmHg. Thus, the maximal colloid dose was 15 mL×kg^−1^×h^−1^. If MAP decreased below 40 mmHg or if the fluid boluses failed to raise MAP over 50 mmHg, an i.v. infusion of noradrenaline was started at 0.03 µg×kg^−1^×h^−1^ with an initial bolus of 0.1 mg. Core temperature was maintained within 37.8–40.2°C with the addition or removal of blankets and fluid warmers.

### Protocol

The animals were given endotoxin (*E.coli*: 0111:B4; Sigma Chemical Co., St Louis, MO, USA) obtained from a single batch, dissolved in 0.9% sodium chloride solution during the entire experiment. To be able to investigate if there were dose differences in the induction of tolerance, the animals were randomized to receive one of two basal endotoxin infusion rates or 0.9% sodium chloride solution alone. Nine animals received a continuous infusion of 0.063 µg endotoxin×kg^−1^×h^−1^ for 24 h, whereas nine received 0.25 µg endotoxin×kg^−1^×h^−1^ for 24 h. Three animals received 0.9% sodium chloride solution and served as controls.

A gradual increase in the endotoxin infusion rate attenuates the initial porcine pulmonary hypertension associated with endotoxin administration [22], [23]. Accordingly, the infusion rate was started at 30% of the target and increased every 5 min to reach the target endotoxin infusion rate after 30 min.

### *Ex vivo* endotoxin stimulation

At baseline and every third hour throughout the experiment, 1.9 mL of blood was sampled. Immediately after the sampling, 0.1 mL of 200 ng×mL^−1^ endotoxin, in the form of purified lipopolysaccharide (LPS) from *E.coli*: 0111:B4 (Sigma Chemical Co., St Louis, MO, USA), was added to each blood sample resulting in a whole blood concentration of 10 ng endotoxin×mL^−1^. Following incubation at 39°C for 2 h, the blood samples were centrifuged, the supernatants transferred to plasma tubes and stored at −30° until analysis.

### Measurements

Arterial blood gases, arterial and mixed venous oxygen saturation and base excess (BE) were analyzed on an ABL™ 5 and Hemoximeter™ (Radiometer, Brønhøj, Denmark). Blood was analyzed on a CELL-DYN 4000™ (Abbott Scandinavia AB, Kista, Sweden) for leukocytes and platelets. Plasma and urine creatinine measurements were performed on an Architect Ci8200 analyzer (Abbott Laboratories, Abbott Park, IL, USA). Commercial porcine-specific sandwich enzyme-linked immune-sorbent assay (ELISA) was used for the determination of TNF-α, interleukin-6 (IL-6) and interleukin-10 (IL-10) in plasma (DY686 (IL-6) and DY690B (TNF-α), R&D Systems, Minneapolis, MN, USA and KSC0102 (IL-10), Invitrogen, Camarillo, CA, USA). The ELISAs had an intra-assay coefficient of variation (CV) of less than 5% and a total CV of less than 10%. Total nitrite concentration in urine was measured, after enzymatic conversion of nitrate to nitrite by nitrate reductase, with the Parameter™ assay (SKGE001, R&D Systems, Minneapolis, MN, USA). The urine samples were diluted 1∶5 prior to the assay according to the recommendations of the manufacturer.

All blood and urine analyses were performed every third hour. Arterial blood gases, MAP, central venous pressure, MPAP, pulmonary capillary wedge pressure and core temperature were registered hourly throughout the study. In addition, blood cell counts were performed hourly for the first six hours. Cardiac output was assessed hourly with the thermodilution method. Cardiac index (CI), left ventricular stroke work index (LVSWI), oxygen consumption (VO_2_), oxygen delivery (DO_2_), static pulmonary compliance and systemic vascular resistance index (SVRI) were calculated from their regular formulas. Airway pressures and respiratory volumes were recorded from ventilator readings. Urine output was registered every hour with the first reading made after the first hour had passed. Nitrate/nitrite excretion in urine was calculated as the product of total nitrite concentration and urine output.

### Calculations and statistics

Primary endpoint was to investigate the temporal development of the propensity to release TNF-α following *ex vivo* endotoxin stimulation by comparing the cytokine values before and after *ex vivo* stimulation. TNF-α, IL-6 and IL-10 concentrations are log-normally distributed [24] and thus, these values were logarithmically transformed. Data were analyzed by repeated measures analysis of variance (ANOVA). In the primary analysis, the group by time interaction in the ANOVA was used to determine whether the *in vivo* and *ex vivo*-stimulated TNF-α levels, after the peak concentrations at 3 h, followed different temporal dynamics over time. IL-6 and IL-10 levels were then analyzed in the same way.

Repeated measures ANOVA was also used in the analysis of the effect of catecholamines and in the analysis of the dose effect of the two endotoxin infusions. If no differences in TNF-α, IL-6 or IL-10 values, neither *in vivo* nor *ex vivo*, between the two doses of endotoxin infusion were found, the values from both dose groups were to be combined.

The difference between TNF-α values, after and before *ex vivo* endotoxin stimulation, ΔTNF-α, was calculated for individual animals at baseline and every third hour. At a given time point, endotoxin tolerance was defined as a significant reduction in ΔTNF-α in comparison with that at 0 h. ΔIL-6 and ΔIL-10 were calculated and analyzed similarly to ΔTNF-α. Since the *ex vivo* response may be limited by the maximal capacity of cytokine production at the time of the *in vivo* peak, the earliest time point for the analysis of endotoxin tolerance was at 6 h. Because log ΔTNF-α, ΔIL-6 and ΔIL-10 did not approximate to normal distribution, Wilcoxon's matched pairs test was used in the comparisons.

The association between endotoxin response at different time points and organ dysfunction and hypoperfusion parameters in individual animals was analyzed by Spearman rank order correlations. Because ΔTNF-α values are inversely related to endotoxin tolerance, negative correlation values represent a positive correlation to endotoxin tolerance and vice versa. Correlation analyses were also employed to analyze the degree of individual responses in ΔTNF-α, ΔIL-6 and ΔIL-10.

Statistica™ (StatSoft, Tulsa, OK, USA) was used in the statistical calculations. A p-value of <0.05 was considered significant, except in the correlation analyses, where p<0.01 (r = 0.59; n = 18) was used because of multiple testing. Values are mean ± SE, unless otherwise stated.

## Results

Eleven animals in the endotoxin groups needed additional fluid boluses according to the protocol, with a median dose of 10 mL×kg^−1^ (range 0–80 mL×kg^−1^). In addition, five of the animals needed vasopressor support. All noradrenaline infusions were started within the first 4 h of the experiment and required throughout the experiment. The *in vivo* and *ex vivo* cytokine values of the animals receiving noradrenaline were compared with those of the other animals. No differences were found, neither for *in vivo* concentrations of TNF-α, IL-6 or IL-10 (p = 0.42, 0.82 and 0.62, respectively), nor for *ex vivo* concentrations (p = 0.49, 0.45 and 0.66, respectively). As to respiratory interventions, 12 animals had their FiO_2_ raised at 24 h (median for all animals 0.43; interquartile (IQ) range 0.31–0.50). Fifteen animals needed adjustments of the V_T_, 4 required an increase, while 11 required a decrease. The median ratio of V_T_ at 24 h to V_T_ at baseline was 0.93 (IQ-range 0.86–1.00). In the control group two animals needed one bolus dose of colloid fluid (10 mL×kg^−1^), whereas no animal required vasopressor support. One animal needed increased FiO_2_ at 24 h (0.45) and all three required adjustments of the V_T_. The median ratio of V_T_ at 24 h to V_T_ at baseline was 0.87 (range 0.82–1.06).

Comparing the two infusion rates, no dose-response relation could be detected at 6 h and later in the *in vivo* TNF-α, IL-6 and IL-10 concentrations (p = 0.91, 0.36 and p = 0.83, respectively) or in the *ex vivo* stimulated concentrations (p = 0.26, 0.92 and 0.53, respectively). According to the statistical plan, values from the two groups were combined in the following analyses.

**Figure 1:**
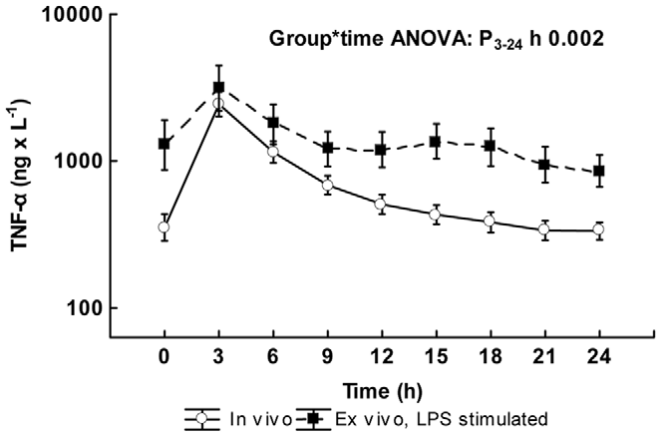
Basal- and *ex vivo*-stimulated TNF-α time concentration curves during the experiment. Values are logarithmically transformed and given as mean ± SE. The log values on the y-axis have been transformed to absolute values. The p-value is the result of the ANOVA for repeated measures group by time interaction analysis comparing the two curves between 3 and 24 h (n = 18). The group analysis as well as the time analysis of the ANOVA for repeated measures comparing the two curves between 3 and 24 h (n = 18), both resulted in p-values of <0.001.

The endotoxin infusion resulted in an increase in TNF-α concentrations with the highest values at 3 h, after which the concentrations gradually returned to normal values (Figure 1). *Ex vivo* endotoxin stimulation from 3 to 24 h resulted in a significant increase in TNF-α values (p<0.001) (Figure 1). In the primary analysis, the *ex vivo* TNF-α values showed different temporal dynamics than the *in vivo* values as indicated by a highly significant group by time interaction analysis comparing the *ex vivo* and the *in vivo* values after the TNF-α peak, i.e. from 3 to 24 h (p = 0.002). This interaction effect was mainly caused by higher *ex vivo* stimulation values from 12 to 21 h.

In the control animals, the *in vivo* TNF-α log concentration was 2.43±0.06 ng×L^−1^ at baseline, corresponding to a concentration of 273 ng×L^−1^. The ensuing values did not change significantly from those at baseline and the mean variation from baseline at each time point was 0.04±0.03 ng×L^−1^. After *ex vivo* endotoxin stimulation, there were marked increases reaching 3.78±0.21 ng×L^−1^ at baseline, corresponding to a concentration of 5997 ng×L^−1^. The *ex vivo* stimulated values did not change significantly from 3 to 24 h, the mean variation from baseline being 0.05±0.04 ng×L^−1^. The group by time interaction did not reach statistical significance.

**Figure 2:**
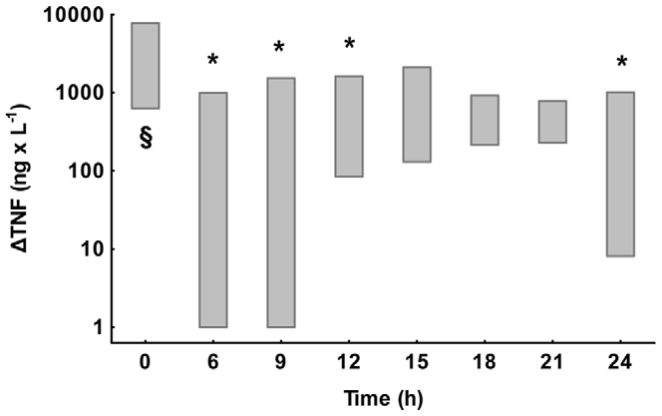
Differences in TNF-α after and before *ex vivo* stimulation, ΔTNF-α, at different time points during the experiment in the animals given endotoxin (n = 18). Values are interquartile ranges. Statistical significance at different time points was tested on the individual changes from time zero using Wilcoxon's matched pairs test. The bar marked with § represents stimulation without prior exposure to endotoxin at 0 h. Bars marked with * represent endotoxin tolerance with a significant reduction of ΔTNF-α in comparison with that at 0 h. Unmarked bars indicate that criteria for endotoxin tolerance were not fulfilled.

The median difference between *ex vivo* and *in vivo* TNF-α concentrations, ΔTNF-α, at time zero was 1410 ng×L^−1^. At 6 h and ensuing hours, ΔTNF-α was lower. The magnitude of this reduction, in comparison with that at time zero, is shown in Figure 2. Significant reductions in ΔTNF-α indicating endotoxin tolerance, was noted at 6, 9, 12 and 24 h (p = 0.004, 0.01, 0.04 and 0.02, respectively). The nadir point of ΔTNF-α, indicating maximal endotoxin tolerance, occurred already at 6 h.

Plasma IL-6 reached a log concentration of 2.64±0.09 ng×L^−1^, corresponding to a mean concentration of 437 ng×L^−1^ at 3 h and was virtually unchanged until 6 h after which concentrations gradually decreased (Table 1). After 3 h, there was no significant increase in IL-6 after *ex vivo* endotoxin stimulation, no significance in the group by time interaction and endotoxin tolerance was noted at all time points after the peak. Plasma IL-10 reached a log peak concentration of 1.54±0.11 ng×L^−1^ corresponding to a mean concentration of 35 ng×L^−1^ at 3 h, after which concentrations decreased (Table 1). In contrast to TNF-α and IL-6, no endotoxin tolerance was observed at any of the time points and ΔIL-10 increased slowly with time. Whereas ΔIL-10 did not correlate to ΔTNF-α at any time point, the peak IL-10 concentration correlated negatively to ΔTNF-α at all of the time points (r = −0.46 to −0.69). In the control animals *in vivo* IL-6 and IL-10 concentrations did not change from those at baseline and after *ex vivo* endotoxin stimulation there were increases that did not change significantly from 3 to 24 h (data not shown).

**Table 1:** Plasma concentrations of IL-6 and IL-10 *in vivo* and *ex vivo* after LPS stimulation at different time points during the experiment.

		0	6	12	18	24
**Plasma IL-6**	**mean±SE (log_10_ ng×L^−1^)**	1.85±0.12	2.65±0.09	2.34±0.10	2.17±0.08	2.12±0.07
	**geometric mean (ng×L^−1^)**	72	443	218	148	131
***Ex vivo*** ** IL-6**	**mean±SE (log_10_ ng×L^−1^)**	2.09±0.14	2.64±0.11	2.38±0.10	2.21±0.06	2.17±0.06
	**geometric mean (ng×L^−1^)**	124	441	241	165	149
**Plasma IL-10**	**mean±SE (log_10_ ng×L^−1^)**	1.14±0.13	1.37±0.11	1.20±0.09	1.05±0.09	1.01±0.09
	**geometric mean (ng×L^−1^)**	14	23	16	11	10
***Ex vivo*** ** IL-10**	**mean±SE (log_10_ ng×L^−1^)**	1.27±0.22	1.37±0.17	1.31±0.15	1.22±0.15	1.33±0.16
	**geometric mean (ng×L^−1^)**	18	23	20	16	21

Values are given as mean±SE and geometric mean, n = 18.

**Figure 3:**
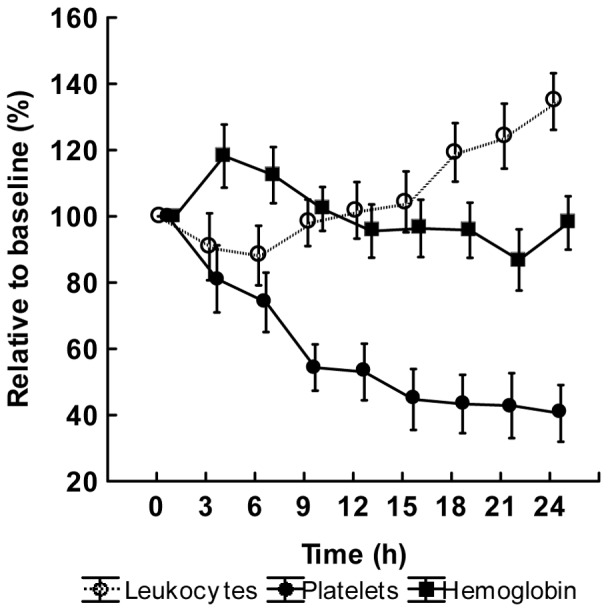
The development during the experiment of leukocyte count, platelet count and hemoglobin. Values are given as relative to the baseline value in percent. Mean ± SE, (n = 18). Baseline values: Leukocyte count 10.6±1.0 10^9^×L^−1^, Platelet count 380±29 10^9^×L^−1^, Hemoglobin 76±2 g×L^−1^.

**Figure 4:**
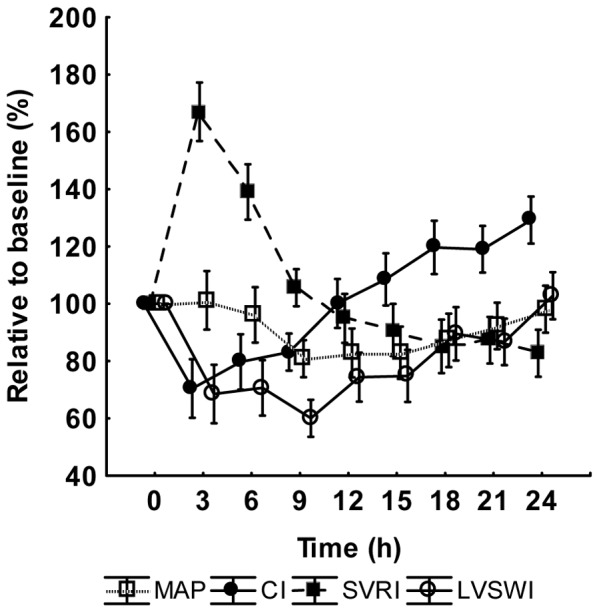
The development during the experiment of left ventricular stroke work index (LVSWI), cardiac index (CI), systemic vascular resistance index (SVRI) and mean arterial pressure (MAP). Values are given as relative to the baseline value in percent. Mean ± SE, (n = 18). Baseline values: LVSWI 48±3 g×m^−2^, CI 4.3±0.3 L×min^−1^×m^−2^, SVRI 1757±127 dyne×s×cm^−5^, MAP 92±4 mmHg.

**Figure 5:**
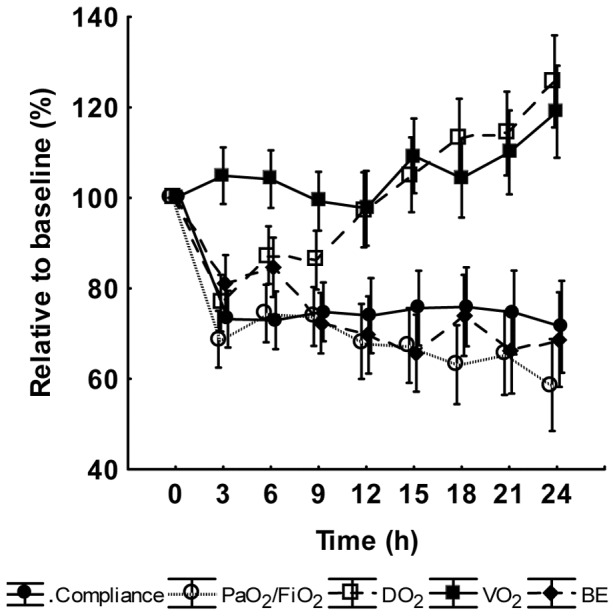
The development during the experiment of compliance, PaO_2_/FiO_2_, oxygen delivery (DO_2_), oxygen consumption (VO_2_) and base excess (BE). Values are given as relative to the baseline value in percent. Mean ± SE, (n = 18). Baseline values: Compliance 25±1 mL×cm H_2_0^−1^, PaO_2_/FiO_2_ 426±16 mmHg, DO_2_ 454±36 mL×min^−1^, VO_2_ 134±8 mL×min^−1^ and BE 7±0.5 mmol×L^−1^.

Levels of leukocytes, platelets and hemoglobin as well as circulatory and organ dysfunction parameters at baseline, except creatinine clearance, are shown as absolute values in the figure legend to Figures 3, 4, and 5, whereas changes over time for the variables are expressed as values relative to baseline in Figures 3, 4, and 5. A biphasic development was observed for hemoglobin and leukocyte count. Hemoglobin increased to a peak at 3 h followed by a slow return to baseline values towards the end of the experiment. The leukocyte count, on the other hand, decreased from 10.5±4.5 10^9^×L^−1^ (mean±SD) at baseline to 7.3±3.8 10^9^×L^−1^ at the nadir point at 2 h, after which there was a gradual increase to values approximately 30% higher than baseline at 24 h. Platelet count showed a continuous decrease during the entire experiment. The transition from an early hypodynamic to a hyperdynamic circulatory state was evident after 12 h. LVSWI followed the same pattern as that of CI, whereas PaO_2_/FiO_2_ and pulmonary compliance declined. BE showed a slight decrease. In creatinine clearance, there was a minor reduction from a median of 130 mL×min^−1^ (IQ range 25–243 mL×min^−1^) to 100 mL×min^−1^ (IQ range 15–218 mL×min^−1^) at 24 h. Median urinary nitrate/nitrite excretion was 38.3 µmol×h^−1^ (IQ range 19.1–109.8 µmol×h^−1^) at baseline. At 3 h there was an increase to 64.5 µmol×h^−1^ (IQ range 44.2–114.6 µmol×h^−1^) that, however, did not reach statistical significance (p = 0.35, *post hoc* Wilcoxon's matched pairs test). The values from 3 to 24 h were relatively stable.

Correlations between values of ΔTNF-α at baseline, at the time of maximal endotoxin tolerance at 6 h and at the end of the experiment with those reflecting different physiological parameters at the end of the experiment are shown in Table 2. The ΔTNF-α value at baseline correlated to that at 6 h and 24 h, indicating individual TNF-α responses in the animals. The same was found for ΔIL-6 and ΔIL-10. ΔTNF-α at 24 h correlated significantly to circulatory variables at the end of the experiment, negatively to CI and positively to SVRI and MAP. In fact, these correlations were observed already with the ΔTNF-α value at the time of maximal endotoxin tolerance, 18 h earlier, whereas no correlations were seen with ΔTNF-α values at baseline (Table 2). At 6 h, ΔTNF-α was also associated with the level of thrombocytes. In contrast to ΔTNF-α, the TNF-α concentration at 6 h did not demonstrate any significant correlations (Table 3). Noteworthy was that ΔTNF-α at 6 h did not correlate to the circulatory variables at that time point, i.e. at 6 h (data not shown). ΔTNF-α did not at any time correlate to the total nitrate/nitrite excretion the last hour of the experiment, Table 2. However, nitrate/nitrite excretion during the early phase at 3 h correlated negatively to ΔTNF-α during the following hours (r = −0.58, −0.51, −0.44, −0.60, −0.44, −0.44, −0.46 at 6 h, 9 h, 12 h, 15 h, 18 h, 21 h and 24 h, respectively).

**Table 2:** Correlation between ΔTNF-α at baseline, at the time of maximal endotoxin tolerance (6 h) and at the end of the experiment and laboratory and physiological parameters at the end of the experiment (n = 18).

	ΔTNF-α baseline	ΔTNF-α 6 h	ΔTNF-α 24 h
**ΔTNF-α baseline**	1.00	0.35	0.65*
**ΔTNF-α 6 h**		1.00	0.80*
**ΔTNF-α 24 h**			1.00
**Leukocyte count**	0.20	0.38	0.35
**Platelet count**	0.37	0.62*	0.58
**Hemoglobin**	0.06	0.47	0.42
**MAP**	0.34	0.59*	0.60*
**Cardiac index**	−0.26	−0.64*	−0.63*
**SVRI**	0.44	0.70*	0.78*
**DO2**	0.01	0.05	0.08
**VO2**	0.05	0.50	0.44
**Base excess**	−0.04	−0.06	−0.06
**LVSWI**	0.05	0.08	0.25
**PaO2/FiO2**	−0.15	−0.01	0.22
**Pulmonary compliance**	−0.15	−0.02	0.24
**Creatinine clearance**	0.16	0.23	0.33
**Nitrate/nitrite excretion**	0.03	0.25	0.20

* denotes a significant correlation coefficient (p<0.01), Spearman rank order correlation analysis.

**Table 3:** Correlation between TNF-α at baseline, at the time of maximal endotoxin tolerance (6 h) and at the end of the experiment and laboratory and physiological parameters at the end of the experiment (n = 18).

	TNF-α baseline	TNF-α 6 h	TNF-α 24 h
**TNF-α baseline**	1.00	0.45	0.51
**TNF-α 6 h**		1.00	0.74*
**TNF-α 24 h**			1.00
**Leukocyte count**	0.05	−0.32	−0.40
**Platelet count**	−0.14	−0.50	−0.37
**Hemoglobin**	−0.40	−0.39	−0.54
**MAP**	−0.39	−0.42	−0.40
**Cardiac index**	0.12	0.24	0.19
**SVRI**	−0.33	−0.48	−0.44
**DO_2_**	−0.14	−0.14	−0.38
**VO_2_**	0.01	−0.14	−0.26
**Base excess**	0.06	−0.08	−0.14
**LVSWI**	−0.29	−0.28	−0.13
**PaO_2/_FiO_2_**	−0.28	−0.17	−0.10
**Pulmonary compliance**	−0.08	−0.25	−0.04
**Creatinine clearance**	−0.36	−0.25	−0.32
**Nitrate/nitrite excretion**	−0.25	−0.17	−0.34

* denotes a significant correlation coefficient (p<0.01), Spearman rank order correlation analysis.

In similarity to ΔTNF-α, ΔIL-6 at 6 h correlated to CI and SVRI, whereas ΔIL-10 did not correlate to any of these parameters (data not shown).

## Discussion

In many experimental *in vivo* models of endotoxin tolerance, repeated single doses of endotoxin have been used to induce tolerance, whereas in many *in vitro* models cells are incubated with endotoxin for times of varying length [4]. In the present investigation the early phase endotoxin tolerance was studied in an *in vivo* model that was designed to be as clinically relevant as possible. A continuous endotoxin load was used both to more closely resemble the clinical situation caused by Gram-negative bacteremia, with a sustained release of endotoxin from infecting bacteria or an increased translocation from the gastrointestinal tract [25], and to achieve a maximal development of endotoxin tolerance. This model has previously been shown to result in a constant plasma endotoxin concentration [19] and, in this aspect, it has similarities to the *in vitro* models. In contrast to these, the first endotoxin-containing medium is not removed before exposing the blood to the second endotoxin challenge. However, the *ex vivo* endotoxin challenge concentration in this study was very high, 10 ng×mL^−1^. Calculated from the previous endotoxin study in which plasma concentrations were measured [19], this *ex vivo* concentration corresponds to more than hundred-fold increases from those *in vivo*. Thus, the *in vivo* plasma concentration must be considered negligible in comparison with that *ex vivo*. Furthermore, in the calculation of the *ex vivo* endotoxin response the basal *in vivo* cytokine concentration was subtracted from that found after the *ex vivo* challenge as has been done in the clinical studies with their varying basal levels [13], [14].

The instrumentation of the animals have similarities to that seen in intensive care patients and to further increase the clinical relevance of this large animal model, goal-directed treatment according to general intensive care principles was applied [26]. However, these measures increase the level of complexity and several of them affect the innate immune system. The animals in this study were allowed to stabilize from the acute physiological responses to the surgical preparations only for 30 min before the experimental protocol was started. This resulted in slight increases in TNF-α baseline values at time zero (Figure 1), whereas those of IL-6 and IL-10 (Table 1) as well as changes in leukocyte count (Figure 3) were limited. The goal-directed protocol allowed for administration of catecholamines that are well known for their suppressing effect on the innate immune system [27]. However, when analysed separately, the levels of the *in vivo* and *ex vivo* cytokines in this study did not differ between animals that received noradrenaline and those that did not. Many anesthetic agents have suppressing effects on the production of inflammatory cytokines, e.g. morphine [28], ketamine [29], propofol [30] and pentobarbital [31]. In this model all anesthetic agents were given in a standardized dose to all animals as a continuous infusion, thus reasonably not influencing the temporal development of endotoxin tolerance. Finally, the animals were ventilated with a medium-high tidal volume, which may affect the innate response but the small adjustments in diverging directions cannot reasonably explain the observed temporal development of tolerance. Thus, the effect of these confounding factors on the results of this study is most probably limited and, most importantly, the control animals subjected to the same regimen did not demonstrate any changes in the response to *ex vivo* endotoxin challenge during the experiment.

Endotoxin tolerance, as indicated by a low TNF-α and IL-6 increase after *ex vivo* stimulation with endotoxin, was observed at different times in the present study. To study the dose response in the development of endotoxin tolerance two endotoxin infusion rates were employed. These doses were selected from a dose-titrating study [24] in which the 0.25 µg endotoxin×kg^−1^×h^−1^ dose was shown to cause significant effects on circulation, hypoperfusion and organ dysfunction, whereas the 0.063 µg endotoxin×kg^−1^×h^−1^ dose resulted in only mild symptoms resembling those observed in human experimental endotoxin studies. Despite major differences in the initial physiological responses (data not shown), no significant dose dependency in the time concentration TNF-α curves could be demonstrated after the peak, neither *in vivo* nor after endotoxin challenge *ex vivo*. This finding indicates that endotoxin tolerance may be marked already after low amounts of endotoxin, which is in agreement with previous human data [32]. In contrast to an undetectable dose effect, there was a significant variation in endotoxin tolerance over time. The strongest suppression of the TNF-α response was seen already at 6 h, i.e. quite early after the initial proinflammatory peak. Contrary to the hypothesis that a continuous endotoxin infusion would lead to a constant suppression of ΔTNF-α, the maximal suppression was followed by a variation of the level of endotoxin tolerance that was highly significant (Figure 1). This effect was mainly caused by the higher TNF-α values after *ex vivo* endotoxin stimulation at 15 to 21 h, which can also be seen in Figure 2, where the criteria for endotoxin tolerance were not fulfilled at these time points. The suppression of ΔIL-6 values was almost total and therefore differences in dynamics difficult to observe. In similarity to TNF-α and IL-6, the concentration of the anti-inflammatory cytokine IL-10 fell after the peak at 3 h, whereas the propensity to increase the production *ex vivo* was not significantly affected at any time point. In this study the development of endotoxin tolerance was more pronounced at higher values of IL-10, which is consistent with previous findings, where IL-10 has been shown to be one of the anti-inflammatory substances that can induce endotoxin tolerance [33]. Whether the trends at 24 h with increasing endotoxin tolerance represent a true biphasic course because of an early anti-inflammatory response that is followed by a transient anti-anti-inflammatory response need further studies, as do the mechanisms underlying this event. However, our results indicate that the time of administration of a second hit is more important than the magnitude of the preexposure dose.

Many cells exhibit changes in the production of inflammatory mediators in a state of tolerance, e.g. macrophages, monocytes, neutrophilic granulocytes, fibroblasts and dendritic cells [34]. In the present study, the leukocytes in whole blood were continuously exposed to endotoxin *in vivo*. Thus, one concern could be that changes in the leukocyte count, induced by the *in vivo* activation by endotoxin, could be responsible for the changes in tolerance during the experiment. However, circulating leukocytes reached their lowest values at 2 h when cytokine production was maximal and at the time for maximal tolerance, they had returned to almost the same values as at time zero. Leukocyte count and endotoxin tolerance then followed different time courses, suggesting that the endotoxin tolerance seen in this study was not associated with the leukocyte count. However, the induction and dynamics of tolerance, as well as the mechanisms involved, are highly complex as has recently been modelled by Fu et al. [34]. Endotoxin tolerance is not solely dependent on reprogramming phenotypes but also on more rapid mechanisms, such as iNOS-induced modulation of tolerance and inhibition of NF-κβ by IL-10 [33].

The individual Δ-values of all three cytokines at baseline and at the time of maximal response correlated strongly to those at the end of the experiment, indicating distinctive individual courses in the cytokine responses over time. Of special interest are the correlations between the hemodynamic parameters and ΔTNF-α and ΔIL-6, relations that can be noted already at the time of maximal endotoxin tolerance at 6 h. The positive correlation to MAP and SVRI and the negative one to CI indicate that the more tolerance to endotoxin the animal displays at the time of maximal tolerance, the lower the SVRI and the higher the CI, i.e. the more hyperdynamic the circulation will be 18 h later. The interventions may, of course, affect outcome parameters but it must be emphasized that changes were made according to a strict protocol resembling clinical practice and without any knowledge of the tolerance results, which would exclude bias in the handling of the animals.

The mechanism behind the association between maximal endotoxin tolerance and circulation remains to be elucidated. Several authors have reported on the roles of inducible nitric oxide-synthetase (iNOS) in modulating endotoxin tolerance [35], [36]. Dias et al. showed that mice deficient of the iNOS gene were not rendered tolerant to endotoxin and that wild-type endotoxin-tolerant mice given the specific iNOS-antagonist aminoguanidine returned to the normal physiological response to endotoxin [36]. One might hypothesize that animals with a high level of tolerance display higher iNOS activity, resulting in a more rapid shift from endotoxin-induced vasoconstriction to an NO-induced vasodilatation and a high CI. Such a possible mechanism is supported by a study by Schmidhammer et al. demonstrating increased nitrite/nitrate levels already at 5 h after the start of endotoxin infusion in pigs subjected to a similar protocol as that in the present study [23]. Furthermore, in a recent study by Wang et al. [37] mouse endothelial cells were, in contrast to other cell types, not rendered endotoxin tolerant following restimulation with endotoxin. Instead, increased gene- and protein expression of proinflammatory cytokines and down-stream adaptors to toll-like receptor 4, the pattern recognition receptor recognizing endotoxin, were found. An increased proinflammatory expression and NO production in the endothelial cells while at the same time other cells express a phenotype of endotoxin tolerance might explain the associations found in this study. Although not reaching statistical significance at all time points, there was an obvious trend towards a negative correlation between early excretion of nitrate/nitrite and ΔTNF-α during the following hours, thus indicating an association between higher NO and increased endotoxin tolerance also in the present study. However, this hypothesis needs further exploration.

Despite endotoxin tolerance being associated with higher CI, there was a trend towards lower VO_2_ in animals with a more pronounced endotoxin tolerance (p<0.05). An increased iNOS activity resulting in a nitric oxide-induced mitochondrial dysfunction might be one reason for such a result [38]. Furthermore, the association between thrombocytopenia and endotoxin tolerance might also be explained by an NO-mediated mechanism, since NO has been shown to be a potent activator of platelets [39].

In contrast to the above-mentioned circulatory parameters, LVSWI, together with the respiratory and renal function parameters, did not correlate to any extent to the individual levels of endotoxin tolerance. At a glance, these results contradict Buttenshoen et al.'s study in which patients with ARDS (PaO_2_/FiO_2_<200) demonstrated a more pronounced endotoxin tolerance than patients with acute lung injury (PaO_2_/FiO_2_<300) [13]. However, recently it has been demonstrated that there is a threshold effect in the pulmonary dose response to endotoxin in endotoxin tolerant animals [20], resulting in a reduced effect at a low challenge dose and an increased pulmonary deterioration at higher challenge doses. This, together with the fact that the plasma level of endotoxin correlated to the severity of the respiratory dysfunction in the study by Buttenshoen et al., might explain the discrepancy.

As has recently been commented, there is lack of information about the time course of the early phase of endotoxin tolerance [18]. The present study has shown that there is a temporal variation of endotoxin tolerance over time and that early endotoxin tolerance is related to ensuing organ dysfunction. This will be of importance in the development of experimental models of septic shock with activated anti-inflammatory mechanisms in order to explore clinical questions aimed at patients suffering secondary sepsis, e.g. secondary to surgery, trauma or primary infection. However, the present study has several limitations. Most important is that only the development of tolerance up until 24 h after the start of infusion was studied. Considering the ΔTNF-α response curve over time and the individual course of the animals, the possibility of a transient anti-anti-inflammatory period that might have an association with the generalized Shwartzman reaction described many years ago [16], [40] needs further investigation. Another limitation is that only continuous infusion was used. It cannot be excluded that the scenario may be different after repeated peaks of endotoxin. Even if that is not a common clinical event, such an administration of endotoxin would add to the mechanistic understanding of the phenomenon of endotoxin tolerance. These questions should be addressed in future studies.

## Conclusions

In this study it was demonstrated that there is a temporal variation of endotoxin tolerance that does not follow changes in plasma TNF-α concentrations and that maximal tolerance was observed very early in the course. The degree of endotoxin tolerance at the time of maximal tolerance was associated with a more marked hyperdynamic circulation and thrombocytopenia 18 h later, whereas cardiac, pulmonary and renal function were not. The knowledge of these effects is of relevance when designing experimental studies exploring effects of endotoxin tolerance and secondary sepsis.
